# Novel alterations in corneal neuroimmune phenotypes in mice with central nervous system tauopathy

**DOI:** 10.1186/s12974-020-01803-7

**Published:** 2020-04-28

**Authors:** Haihan Jiao, Laura E. Downie, Xin Huang, Mengliang Wu, Sara Oberrauch, Ryan J. Keenan, Laura H. Jacobson, Holly R. Chinnery

**Affiliations:** 1 Department of Optometry and Vision Sciences, The University of Melbourne, Parkville, Australia; 2 Innate Phagocytosis Laboratory, Florey Institute of Neuroscience and Mental Health, Parkville, Australia; 3 Department of Pharmacology and Therapeutics, The University of Melbourne, Parkville, Australia; 4 Sleep and Cognition Laboratory, Florey Institute of Neuroscience and Mental Health, Parkville, Australia

**Keywords:** Central nervous system tauopathy, Immune cells, Cornea, Sensory nerves, Peripheral nervous system

## Abstract

**Background:**

Tauopathy in the central nervous system (CNS) is a histopathological hallmark of frontotemporal dementia (FTD) and Alzheimer’s disease (AD). Although AD is accompanied by various ocular changes, the effects of tauopathy on the integrity of the cornea, which is densely innervated by the peripheral nervous system and is populated by resident dendritic cells, is still unknown. The aim of this study was to investigate if neuroimmune interactions in the cornea are affected by CNS tauopathy.

**Methods:**

Corneas from wild type (WT) and transgenic rTg4510 mice that express the P301L tau mutation were examined at 2, 6, 8, and 11 months. Clinical assessment of the anterior segment of the eye was performed using spectral domain optical coherence tomography. The density of the corneal epithelial sensory nerves and the number and field area of resident epithelial dendritic cells were assessed using immunofluorescence. The immunological activation state of corneal and splenic dendritic cells was examined using flow cytometry and compared between the two genotypes at 9 months of age.

**Results:**

Compared to age-matched WT mice, rTg4510 mice had a significantly lower density of corneal nerve axons at both 8 and 11 months of age. Corneal nerves in rTg4510 mice also displayed a higher percentage of beaded nerve axons and a lower density of epithelial dendritic cells compared to WT mice. From 6 months of age, the size of the corneal dendritic cells was significantly smaller in rTg4510 compared to WT mice. Phenotypic characterization by flow cytometry demonstrated an activated state of dendritic cells (CD86^+^ and CD45^+^ CD11b^+^CD11c^+^) in the corneas of rTg4510 compared to WT mice, with no distinct changes in the spleen monocytes/dendritic cells. At 2 months of age, there were no significant differences in the neural or immune structures between the two genotypes.

**Conclusions:**

Corneal sensory nerves and epithelial dendritic cells were altered in the rTg4510 mouse model of tauopathy, with temporal changes observed with aging. The activation of corneal dendritic cells prior to the gradual loss of neighboring sensory nerves suggests an early involvement of corneal immune cells in tau-associated pathology originating in the CNS.

## Introduction

Alzheimer’s disease (AD) and tau-related variants of frontotemporal dementia (FTD) are neurodegenerative diseases characterized by the pathological accumulation of tau in the central nervous system (CNS). The accumulation of hyperphosphorylated tau, concomitant with the progressive formation of neurofibrillary tangles (NFTs), further drives impairment of cellular trafficking [1], synaptic dysfunction [2, 3], cognitive deficits [4], and neuronal loss [5, 6]. The effect of CNS degeneration on ocular health [7], such as retinal nerve fiber thinning and retinal neuronal loss, has been described both in patients [8–10] and mouse models of tauopathy [11–13]. As one of the most highly innervated tissues of the body, the cornea has recently been used in clinical studies to non-invasively examine peripheral nervous system pathology secondary to CNS neurodegeneration in vivo [14–20]. However, there is a paucity of preclinical studies aiding the elucidation of causative factors behind corneal changes in neurodegenerative disorders.

The cornea is supplied by sensory nerve axons that form part of the peripheral nervous system (PNS) [21, 22]. A rich supply of sensory nerves originates from the ophthalmic branch of the trigeminal nerve and ramifies to form a plexus in the sub-basal region of the corneal epithelium. Nerve axons branching from the sub-basal nerve plexus (SBNP) extend processes, which ramify and terminate through the entirety of the epithelium where they form the superficial nerve terminals (SNT) [23]. Corneal nerves provide trophic support to maintain both the homeostasis of the corneal epithelium and functional integrity of the ocular surface [24]. Among the resident immune cells, epithelial dendritic cells (DCs) predominantly reside in the basal epithelium of the human [25, 26] and mouse cornea [27, 28], where they not only serve as immune sentinels [29, 30] but also act as a bridge between the innate and adaptive immune systems [31]. Most DCs in the corneal epithelium are located peripherally, with a decline in numbers centrally [26]. Growing evidence shows the involvement of resident corneal DCs in maintaining the homeostasis of corneal nerves [32], suggesting a direct interaction between the immune system and peripheral nerves at the ocular surface.

Corneal epithelial DCs and nerves can be visualized and quantified by corneal confocal microscopy, which is a non-invasive ophthalmic imaging tool [33, 34]. Emerging evidence indicates a decline in the corneal nerve fiber density in patients with Parkinson’s disease (PD) [35, 36], small fiber neuropathy [17] and Multiple Sclerosis (MS) [14, 15]. Recently, reduced corneal sensitivity and altered tear production were reported in individuals with Alzheimer’s disease (AD) [37]. Corneal nerve fiber loss has been shown in patients with mild cognitive impairment (MCI) and dementia, with a strong correlation evident between corneal nerve fiber loss and decreasing cognitive function [16]. However, the precise changes that occur to the neuroimmune interactions between corneal nerves and resident DCs are still unknown in the context of CNS degenerative diseases.

In order to understand the peripheral manifestations of CNS degeneration at the ocular surface, we characterized the temporal effect of CNS tauopathy on the integrity of corneal nerves and resident DCs using the rTg(tauP301L)4510 mouse model of tauopathy [4]. The rTg4510 model overexpresses doxycycline-repressible human mutant tau with the *MAPT* P301L mutation, which is associated with genetic forms of FTD-tau, under the control of the Ca^2+^-calmodulin-dependent protein kinase II (CaMKII) promoter [4]. It is important to note that these random insertions led to disruptions in a number of endogenous genes [38], which exacerbate phenotypic changes in rTg4510 [39]. Nonetheless, among the models of tauopathy, the hTau in these mice promotes progressive age-related NFTs, neuronal loss concomitant with the tau accumulation in the forebrain and hippocampus, followed by the substantial neurodegeneration [4, 6, 40–43], reminiscent of tauopathy in patients with FTD-tau [44]. In this animal model, we report that both peripheral nerves in the cornea and epithelial DCs were altered in mice with the age-related accumulation of pathological tau. Corneal DC morphology was affected prior to corneal nerve degeneration, suggesting that DCs may be involved in the peripheral nerve abnormalities in the presence of CNS tauopathy. This study implicates the potential utility of using corneal neuroimmune phenotypes as landmarks to identify peripheral neuropathology secondary to the CNS degeneration.

## Methods

### Animals

Male and female wild type (WT) and tau transgenic littermates (rTg4510) were bred and housed under specific pathogen-free conditions at the Florey Institute of Neuroscience and Mental Health. Age-matched WT and rTg(tauP301)4510 mice were examined at 2, 6, 8, and 11 months of age (*n* = 6–8 per group per age). All animal procedures were approved by the Animal Ethics Committee at the Florey Institute of Neuroscience and Mental Health and complied with the ARVO Statement for the Use of Animals in Ophthalmic and Vision Research.

### Spectral domain optical coherence tomography

The anterior segment was examined using SD-OCT to ensure there were no clinical signs of inflammation (i.e., inflammatory cells, corneal edema, corneal opacity, or epithelial erosions) or other structural abnormalities in the anterior segment of the eye. Euthanized mice were placed on the animal imaging platform and rodent alignment stage (AIM-RAS) attached to an SD-OCT imaging device (Bioptigen Envisu R22200 VHR; Bioptigen, Inc., Durham, NC, USA). Volumetric 3 mm × 3 mm rectangular scans of the anterior segment were captured using an OCT device with an 18-mm telecentric lens. Central corneal thickness was determined by measuring the distance from the tear film to the endothelium using the ImageJ software (http://imagej.nih.gov/ij/; National Institutes of Health, Bethesda, MD, USA), as previously described [45].

### Wholemount immunofluorescence and confocal microscopy

Corneas from age-matched WT and rTg4510 mice were collected at 2, 6, 8, and 11 months of age and fixed in 100% methanol for 1 h at −20 °C. After three washes in PBS, corneas were permeabilized in 20 mM EDTA for 40 min at 37 °C and then blocked in PBS containing 3% BSA-PBS and 0.3% Triton X-100 for 1 h at room temperature. Tissues were stained using a combination of primary antibodies to identify nerves (rabbit α-β-tubulin III, 1:500, Sigma T2200, St Louis, MO, USA) and DCs (rat α-CD45, 1:500, BD Biosciences, Franklin Lakes, NJ, USA). The primary antibody incubation was kept at 4 °C overnight and the corneas were washed and incubated with corresponding secondary antibodies goat α-rabbit 647 and goat α-rat Cy3 (1:1000, Invitrogen, Carlsbad, CA, USA) for 2 h at room temperature. Corneas were examined with a fluorescence microscope (Olympus BX511, Zeiss) to measure dendritic cell density and morphology, and with a confocal microscope (Leica TCS SP8; Leica, Germany) to visualize corneal nerves.

Confocal Z-projections were generated for the SBNP and SNT. Area thresholds, which measure the percentage field area occupied by corneal nerves, were quantified separately for the SBNP and SNT in the central and peripheral corneal regions, as previously described [46, 47]. All image analyses were carried out in a masked fashion, with the genotypes unmasked after the data acquisition for each age cohort. Corneal nerve beading was measured using the Z-stacked images of the SBNP. A series of binary conversions were performed using the default method of thresholding in Image J ("Auto-Threshold"). The image was further processed to create the binary images (total nerve projections, continuous and beaded) for each confocal image using 'Subtracting Background' and "Shape Filter". The percentage of 'nerve beading' was calculated (raw integrated density of "nerve beads"/full nerve length × 100) for the central and peripheral cornea. DC morphometric analyses were carried out on images of CD45^+^ DCs using × 10 objective (600 μm × 900 μm). One field from the central cornea and two fields from the peripheral cornea were used for image analysis (DC cell density and morphometric parameters). DC morphometric analyses included DC field area, cell area, total dendrite length (TDL), and number of dendrites per cell using the established protocol in humans [48, 49] and mice [50, 51]. In brief, each cell of interest, isolated by a ROI, was processed through a local threshold and skeletonized, for the cell area and TDL analysis, respectively (Fig. 3). Each parameter had a total of 20–30 cells included for the peripheral cornea and 1–3 cells for the central cornea. Each datapoint on the graph represents the mean for an individual mouse.

### Quantitative real-time polymerase chain reaction (qPCR)

Corneas were stabilized in RNAlater solution overnight at 4 °C before being dissected and processed for RNA extraction (*n* = 11 per genotype) using PureLink RNA Mini Kit (Invitrogen, Life Technologies) according to manufacturer’s instructions. Each sample containing two corneas per mouse was homogenized in lysis buffer containing β-mercaptoethanol using QIAGEN TissueLyser II. PureLink DNase was added to each spin column containing the resulting RNA to prevent contamination. RNA concentration and quality were assessed by ND-2000 spectrophotometer (Nanodrop technologies, USA) for each sample and stored at −80 °C. cDNA was prepared from 500 ng of each RNA sample using Tetro cDNA Synthesis Kit (Bioline, London, UK) according to manufacturer’s protocol.

Gene expression changes were measured via qPCR using Taqman assays for *Gapdh* (Mm99999915_g1, Entrez Gene ID: 131530) and *CaMKIIa* (Mm00437967_m1 Entrez Gene ID: 304088) and Taqman Gene Expression Master Mix (Thermo Fisher Scientific). Each qPCR (duplicates) was run using the QuantStudio 12 K Flex software on the Vii7A Real-Time PCR system at the Melbourne Brain Centre (Applied Biosystems, USA). Analysis was performed using the 2 − ΔΔCt method, which was normalized to the expression of the housekeeping gene.

### Flow cytometry of corneas and spleens

Corneas from 9-month-old WT and rTg4510 mice (*n* = 6 per genotype) were excised and pooled (4 corneas, 2 animals per tube, *n* = 3). Excised corneas were digested in sodium medium containing 2 mg/mL collagenase D (Roche, Indianapolis, IN) and 0.5 mg/mL DNase I (Roche, Indianapolis, IN) for 30 min at 37 °C. Repetitive trituration was performed after the digestion using a sterilized glass Pasteur pipette. Single cell suspensions were filtered through a 70 μm mesh (BD Biosciences) and centrifuged at 4 °C, 600*g* for 5 min. The pellet was resuspended in sodium medium [52] (145 mM NaCl, 5 mM KCl, 0.1 mM Ca^2+^, 10 mM HEPES, 0.1% BSA, 5 mM d-glucose pH 7.5) containing CD16/32 receptor block to avoid non-specific staining. Flow cytometry antibodies, including CD11b-PE, CD45-FITC, and CD11c-PeCy7 (1:400, BD Biosciences, Franklin Lakes, NJ, USA) along with isotype control antibodies, were used to isolate DCs from the mouse corneas, while CD86, MHC-class II, and CD80 all conjugated to allophycocyanin fluorophore (APC) were used to separately visualize the activation status of the DCs. The staining process for the aliquoted single cell suspension was performed on an orbital shaker at 4 °C for 30 min. Unstained controls omitting the above antibodies were also included to confirm the positive and negative cell population. Following the staining, the cells were washed twice in Dulbecco’s phosphate-buffered saline (DPBS), centrifuged at 4 °C, 600*g* for 5 min and resuspended in sodium medium. The single-cell suspension was assessed on CytoFlex S Flow Cytometer (Beckman Coulter). UltraComp eBeads Compensation Beads (Invitrogen, 01-2222-41) and single-stained corneal samples were used to establish and validate compensation gates for the above antibody combinations. The FlowJo software (v10) was used to analyze the data in both genotypes.

### Statistical analyses

All statistical analyses were performed using Prism 7 (GraphPad Software, CA, USA). Statistical significance was assessed using a one-way ANOVA or two-way ANOVA (as appropriate). Unpaired Student's *t-*tests were performed to compare WT and rTg4510 mice for corneal thickness, while a two-way ANOVA was performed to compare the corneal cellular characteristics (nerve and DC parameters in the different age groups). Tukey’s post hoc tests were applied where multiple statistical comparisons were performed. Unpaired Student's *t* tests were performed to assess the comparisons at each age group. An alpha of 0.05 was used to define statistical significance.

## Results

### Structural integrity in the anterior segment of rTg4510 mice

**Fig. 1:**
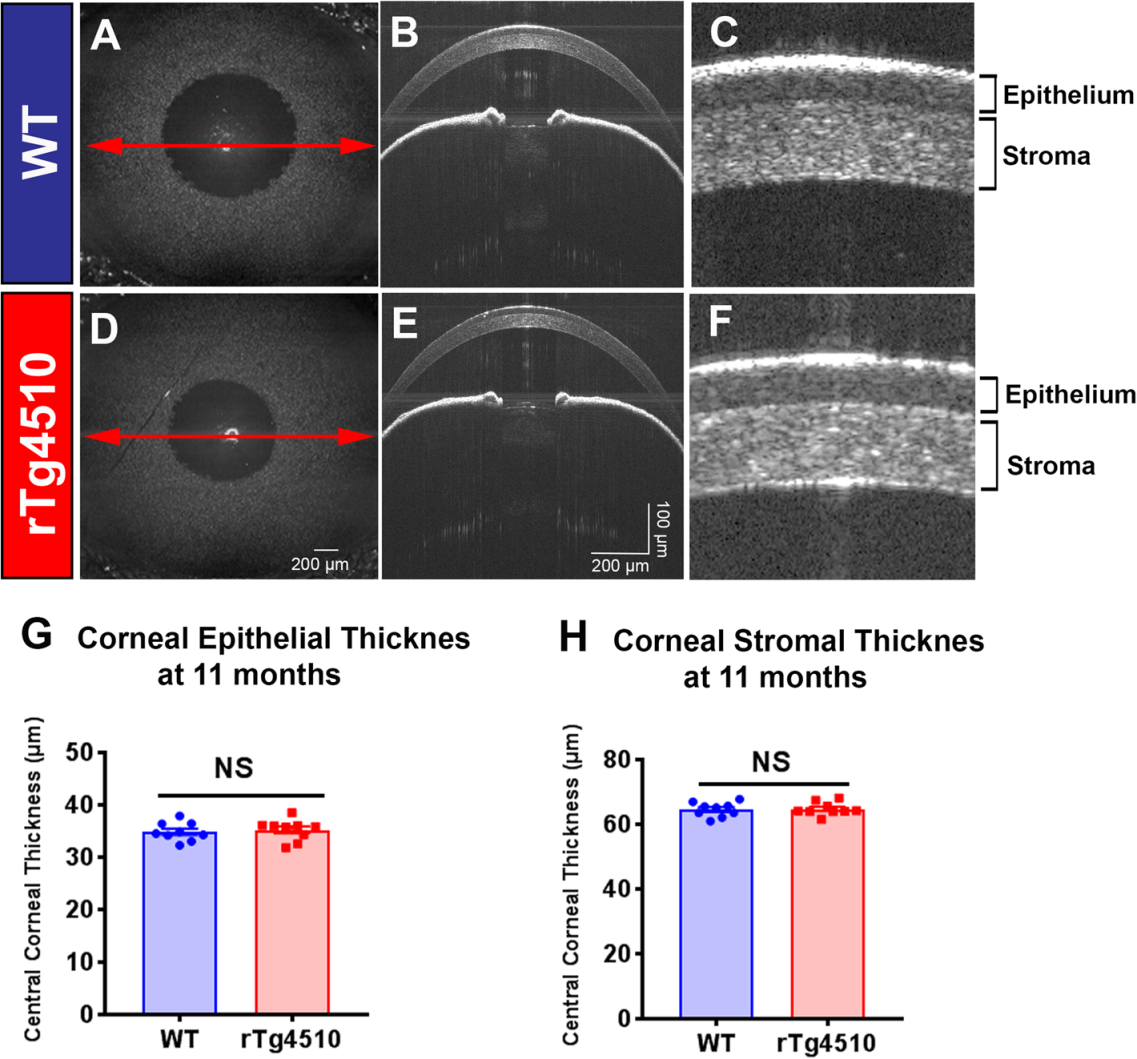
Spectral domain optical coherence tomography (SD-OCT) images of the anterior segment from wild type mice and rTg4510 mice aged 11 months. **a***En face* view of the WT mouse eye (3 mm × 3 mm rectangular scan) and **b** corresponding SD-OCT scan of the anterior segment. Red arrowed line represents the *en face* location of the scan. **c**, **f** Structure of the corneal epithelium and stroma, as shown using SD-OCT. No visible differences in the corneal structure or anterior segment were observed between WT (**a-c**) and rTg4510 mice (**d-f**). **g**, **h** There were no quantitative differences in corneal epithelial (**g**) or stromal thickness (**h**) between WT and rTg4510 mice. Data are shown as mean ± SEM where NS represents a comparison that is not statistically significant (*n* = 8 per group) as assessed using unpaired Student's *t-*test. Scale bars represent 200 μm in Fig. 1**a** and **d**, and 100 μm (vertical axis), and 200 μm (horizontal axis) in Fig. 1**b** and **e**

The anterior segment of WT and rTg4510 mice aged 11 months were examined using SD-OCT to determine if CNS tauopathy was associated with any overt structural or cellular corneal pathology (Fig. 1). There were no structural abnormalities observed in the anterior segment (i.e., cellular infiltrates, epithelial disruption, stromal edema) of rTg4510 compared to WT mice (Fig. 1a–f). There were also no significant differences in central corneal epithelial thickness (Fig. 1g, *P* > 0.05) or stromal thickness in rTg4510 mice compared to WT (Fig. 1h, *P* > 0.05).

### Disrupted corneal nerve architecture in rTg4510 mice

**Fig. 2:**
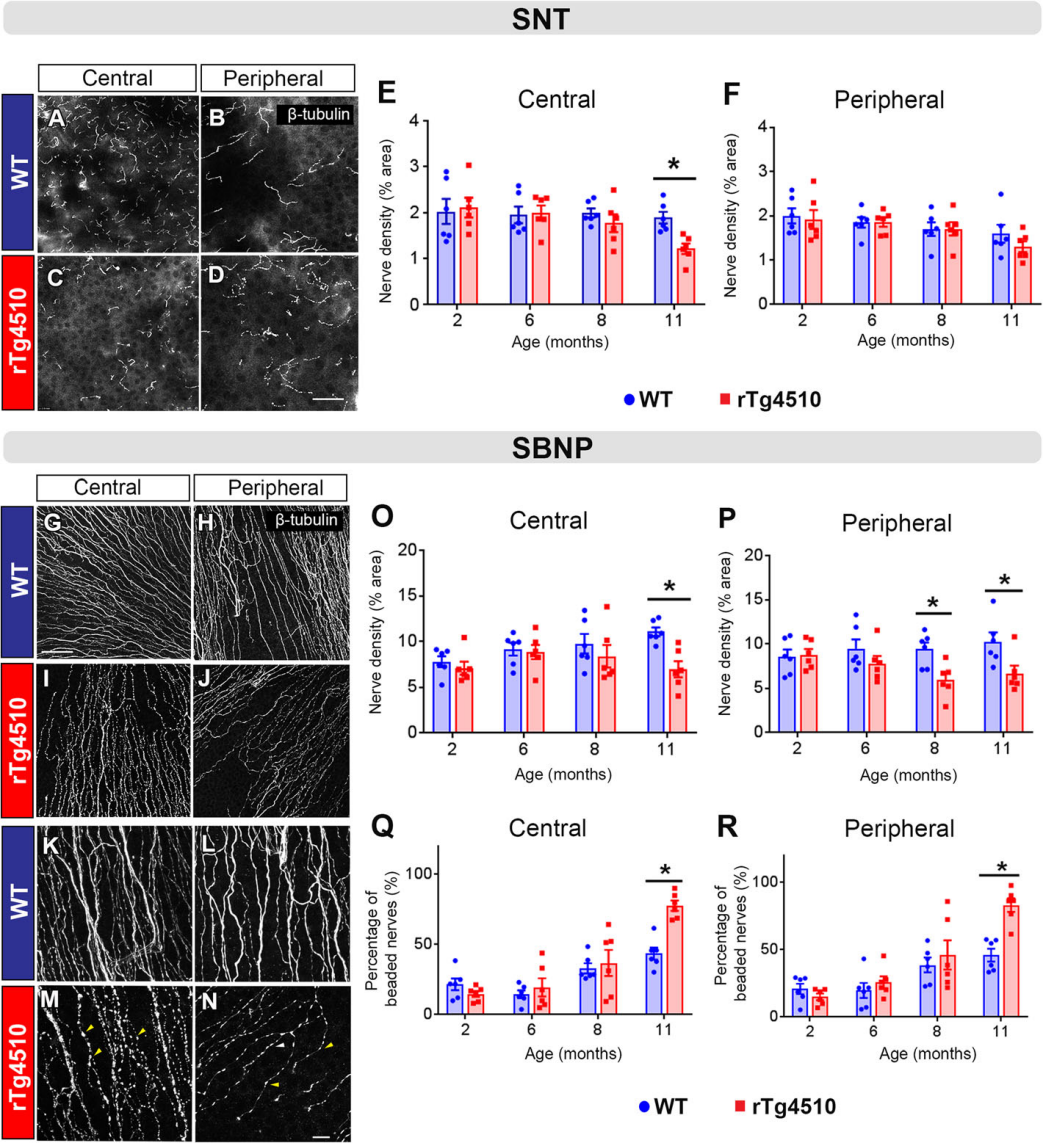
Regional distribution, density and morphology of corneal sensory nerves in WT and rTg4510 mice at 2, 6, 8, and 11 months. **a**–**d** Representative images of the superficial nerve terminals (SNT) in the corneas of WT and rTg4510 mice at 11 months. β-tubulin III^+^ SNTs can be abundantly seen in the central and peripheral cornea of WT mice (**a**, **b**), while rTg4510 mice had a relatively sparse distribution in the central cornea but were spared in the peripheral cornea (**c**, **d**). **e**, **f** Percentage area demonstrates a significantly lower density of SNTs in the central cornea of rTg4510 compared to WT mice at 11 months (*P* < 0.05), while the peripheral cornea showed no significant intergroup difference across the age groups (*P* > 0.05). **g**–**n** Representative images of the sub-basal nerve plexus (SBNP) in corneas of WT and rTg4510 mice showing a lower abundance of SBNPs that appeared in a beaded morphology in the central and peripheral areas of rTg4510 mouse corneas (**m**, **n**; yellow arrowheads). **o**, **p** The density of SBNPs was significantly lower in the central cornea of rTg4510 compared to WT mice at 11 months (*P* < 0.05), while the lower SBNP density occurred in the peripheral cornea from 8 months and persisted to 11 months (*P* < 0.05). **q**, **r** The extent of nerve beading that characterizes the morphology of SBNPs was analyzed; the percentage of beaded nerves was remarkably higher in both central and peripheral regions of rTg4510 mouse corneas at 11 months (*P* < 0.05) but not in the younger age cohorts (*P* > 0.05). Data are shown as mean ± SEM, where * indicates *P* ≤ 0.05 (*n* = 6 per group) as demonstrated using two-way ANOVA and Tukey’s multiple comparisons. Scale bars represent 100 μm for all images except for **f**–**n** where the scale bars are 10 μm

Corneal sensory nerve density and morphology were assessed in WT and rTg4510 mice aged 2, 6, 8, and 11 months using β-tubulin wholemount immunostaining. In the *superficial epithelial layer*, there was a higher density of SNTs (Fig. 2a, b) in the central corneas of WT mice compared to rTg4510 mice (Fig. 2c, d) at 11 months of age. Area threshold analysis confirmed that the density of the SNTs, in the central cornea, was significantly lower in rTg4510 mice compared to WT, at 11 months (Fig. 2e, *P* < 0.05), but not at 2, 6, or 8 months of age (Fig. 2e, *P* > 0.05 at each time point). In the peripheral region, there was no significant difference in the density of the SNTs between rTg4510 and WT mice across the age groups (Fig. 2f, *P* > 0.05).

In the *basal epithelial layer*, the density of β-tubulin^+^ nerve axons forming the SBNP was lower and appeared to demonstrate a highly beaded morphology in both the central and peripheral cornea of rTg4510 compared to WT mice at 11 months of age (Fig. 2g–n). The density of β-tubulin^+^ nerve axons was lower in both central corneal (11 months) and peripheral corneal regions (8 and 11 months) of rTg4510 compared to WT mice (Fig. 2o, p, *P* < 0.05 for both comparisons). Additionally, the nerve axons in both corneal regions displayed a significantly higher proportion of nerve beading in rTg4510 compared to WT mice at 11 months of age (Fig. 2 q, r, *P* < 0.05).

### Altered morphology and distribution of epithelial dendritic cells in rTg4510 mice

**Fig. 3:**
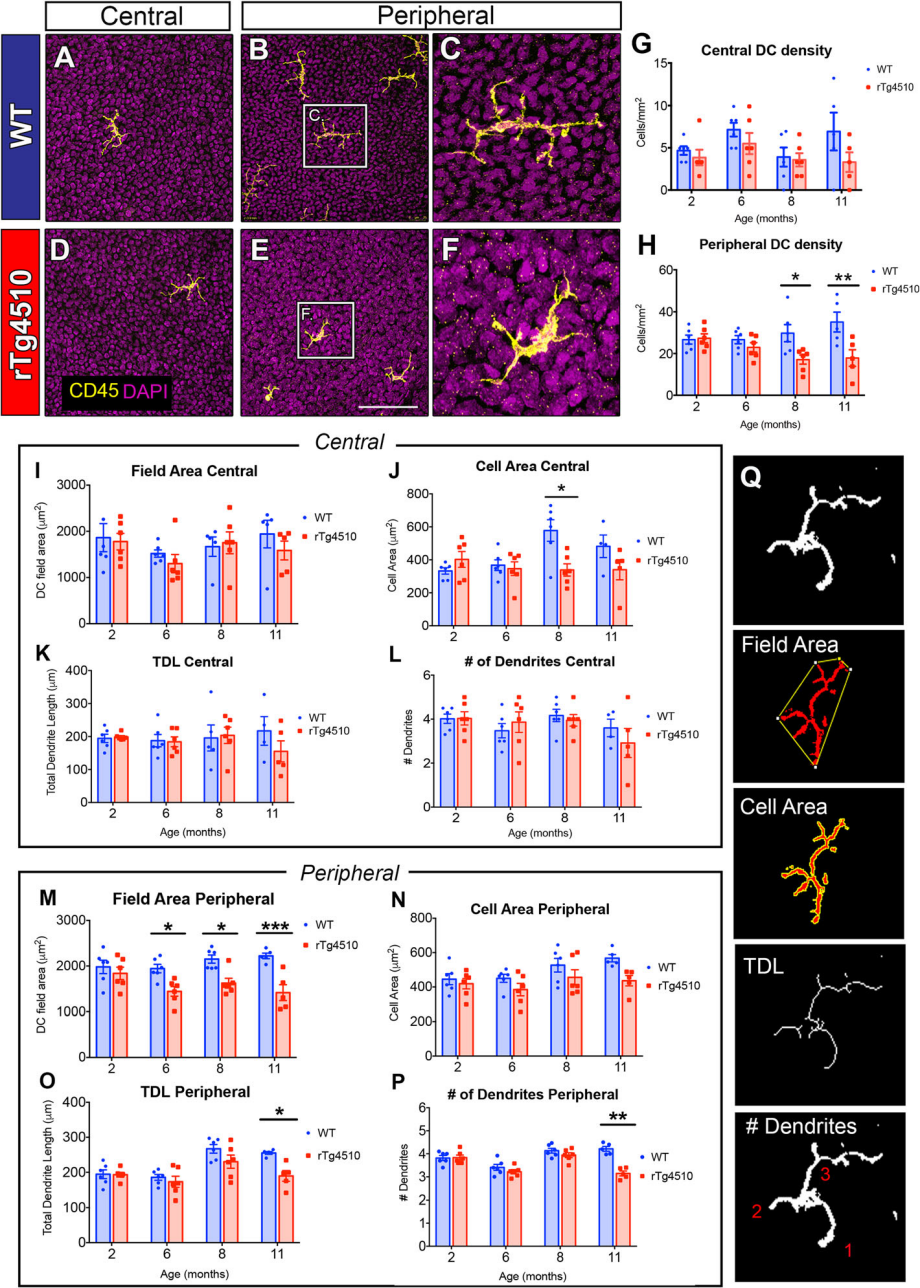
Dendritic cells in the central and peripheral corneal epithelium of WT and rTg4510 mice at 2, 6, 8, and 11 months. **a**–**f** Representative images of CD45^+^ DCs show higher abundance in the peripheral versus central cornea for both genotypes, with few DCs across the peripheral area in rTg4510 mice at 11 months (**d**–**f**). **c**, **f** In rTg4510 mice, CD45^+^ DCs had an altered, amoeboid morphology compared to WT at 11 months. **g** The central cornea showed no significant inter-group differences at any age (*P* > 0.05). **h** Substantial differences were observed in the peripheral cornea, whereby there was a significantly lower DC density in rTg4510 mice compared to WT mice, at both 8 and 11 months (*P* < 0.05). **i**–**p** DCs were analyzed for field area, cell area, total dendrite length (TDL), and number of dendrites per cell. There was no genotype difference in the central cornea except for a larger cell area in the 8-month-old WT mice compared to rTg4510 mice (**j**, *P* < 0.05). **m**–**p** In the peripheral cornea, DC field areas were significantly smaller in rTg4510 mice compared to WT, at 6, 8, and 11 months of age (**m**, *P* < 0.05). Cell area analysis shows no significant genotype difference (**n**). TDL and number of dendrites per cell were less in the 11-month-old rTg4510 mice compared to WT (**o**, **p**; *P* < 0.05). **q** Morphometric methods for field area, cell area, TDL, and number of dendrites per cell. Scale bars represent 100 μm. Date are shown as mean ± SEM, where * indicates *P* ≤ 0.05, **P ≤ 0.01, ***P ≤ 0.001 as determined using the two-way ANOVA and Tukey’s multiple comparisons

Corneal epithelial DCs were visualized by CD45 immunohistochemistry. CD45^+^ cells were visible in the central and peripheral corneal epithelium of WT (Fig. 3a–c) and rTg4510 mice (Fig. 3d–f). There was no significant difference in DC density between WT and rTg4510 mice in the central cornea at 2, 6, 8, and 11 months of age (Fig. 3g, *P* > 0.05 for each comparison). In the peripheral corneal epithelium, DC density was significantly lower in rTg4510 mice aged 8 and 11 months compared to WT mice (Fig. 3h, *P* < 0.05 at both time points). DC morphometric parameters (DC field area, cell area, TDL, and number of dendrites per cell) used clinically as an indicator of DC “maturity” in the corneal epithelium [48] revealed no significant intergroup differences in the central cornea (Fig. 3i–l), except for a higher cell area in the 8-month-old WT mouse corneas compared to rTg4510 mice (Fig. 3j, *P* < 0.05). In the peripheral cornea, DC field area was significantly smaller in rTg4510 mice at 6 months of age, and this small field area persisted at 8 and 11 months (Fig. 3m, *P* < 0.05). Cell area did not differ between WT and rTg4510 for any age group (Fig. 3n, *P* > 0.05). TDL and number of dendrites per cell were substantially lower in the 11-month-old rTg4510 mouse corneas compared to the aged-matched WT (Fig. 3o, p, *P* < 0.05).

### Phenotypic analysis of corneal DCs in the presence of tauopathy

**Fig. 4:**
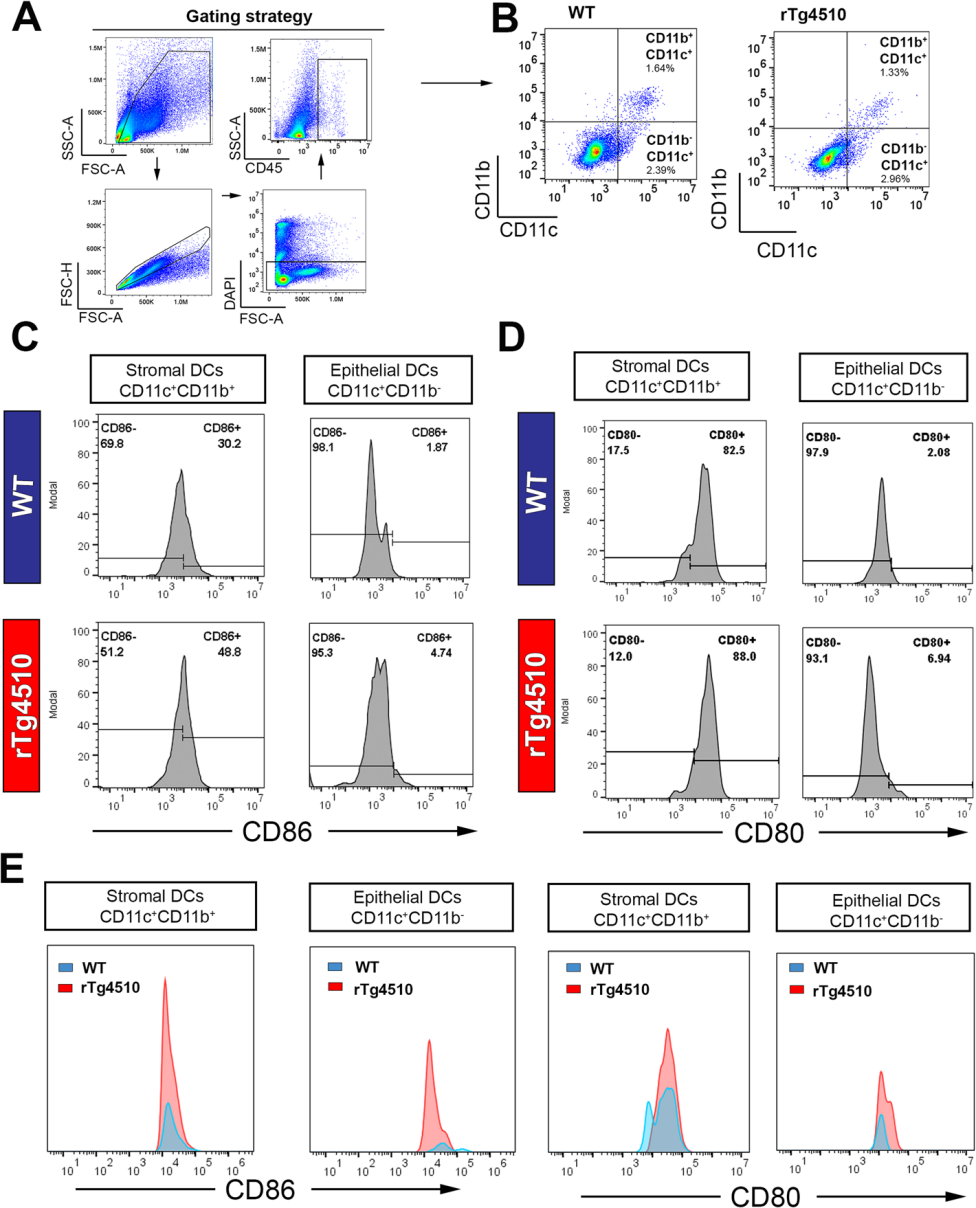
Representative flow cytometric plots showing different populations of dendric cells in the cornea of WT and rTg4510 mice at 9 months of age. **a**, **b** Gating strategies for enumeration of CD45^+^CD11c^+^ CD11b^+^ stromal DCs and CD45^+^CD11c^+^ CD11b^−^ epithelial DCs in WT and rTg4510 mice. **c** Frequencies of CD86^+^ stromal and epithelial DCs in two genotypes. rTg4510 mice had a higher proportion of CD86^+^ stromal DCs (48.8%) compared to WT (30.3%). Proportion of CD86^+^ epithelial DCs were also substantially higher in rTg4510 (4.74%) compared to WT (1.87%). **d** Proportion of CD80^+^ stromal DCs was 82.5% in WT and 88.% in rTg4510. Epithelial DCs, however, comprise a higher proportion of the CD80^+^ population in rTg4510 (6.94%) than WT (2.08%). **e** The corneas from rTg4510 mice had a higher percentage of CD86^+^ epithelial DCs and stromal DCs compared to WT as well as higher percentage of CD80^+^ epithelial DCs and stromal DCs at 9 months of age

Previous flow cytometry studies of the mouse cornea have demonstrated that resident epithelial DCs are CD11c^+^CD11b^−^, whereas the stromal DCs are CD11c^+^CD11b^+^ [53, 54]. Using this paradigm to quantitatively assess corneal DC phenotype, we next investigated the effect of tauopathy on the immunophenotype of corneal DC populations. Here, we adopted a similar gating strategy to compare the activation status of CD11c^+^CD11b^−^ “epithelial” DCs and CD11c^+^CD11b^+^ “stromal” DCs in WT and rTg4510 mice at 9 months of age (Fig. 4a, b). While the total frequency of each population was not markedly different between WT and rTg4510 mice (Fig. 4b), the proportion of CD86^+^ cells was substantially higher in CD11c^+^CD11b^−^ epithelial DC and CD11c^+^CD11b^+^ stromal DC populations of rTg4510 mice (stromal, 48.8%; epithelial, 4.74%) compared to WT mice (stromal, 30.2%; epithelial, 1.87%) (Fig. 4c). The percentage of CD11c^+^CD11b^−^ CD80^+^ “epithelial” DCs was also higher in the rTg4510 mouse corneas (epithelial, 6.94%) compared to WT mice (epithelial, 2.08%) (Fig. 4d). Flow cytometric plots of absolute numbers show that the epithelial DC had a higher number of CD86^+^ and CD80^+^ population in rTg4510 mouse corneas compared to WT (Fig. 4e). The stromal DCs also demonstrated a higher number of CD86^+^ and CD80^+^ cells in the corneas of rTg4510 mice compared to WT (Fig. 4e). These data suggest an activated phenotype of corneal DC subsets in this mouse model of tauopathy.

### Phenotypes of splenic immune cells in rTg4510 mice

**Fig. 5:**
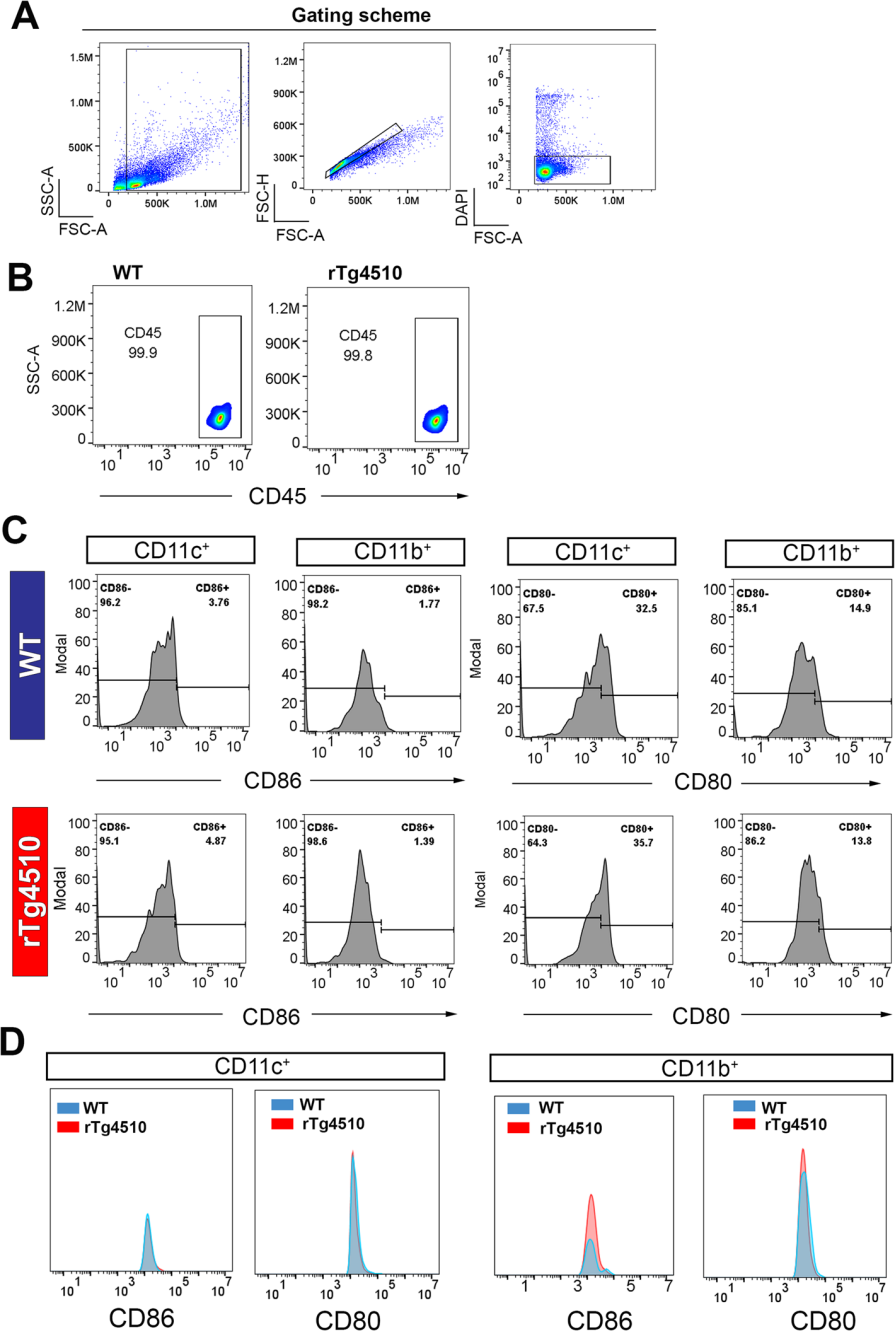
Spleen flow cytometric plots showing the phenotypes of CD45^+^ cell populations in WT and rTg4510 at 9 months. **a** Gating scheme for splenocytes isolation showing DAPI-population was used for the downstream analysis. **b** Total CD45^+^ cells were not different between WT (99.9%) and rTg4510 mice (99.8%). **c** The proportion of CD86^+^ and CD80^+^ population was low in WT and displayed no substantial changes in rTg4510 mice. **d** The absolute number of CD86^+^ and CD80^+^ showed no difference in the CD11c^+^ splenocytes between the two genotypes, but a higher number of CD86^+^ and CD80^+^ cells in the CD11b^+^ splenic immune subset of rTg4510 mice compared to WT mice

To interrogate whether the presence of systemic inflammation accompanied the altered corneal DC phenotype, we analyzed the frequency and activation profile of CD45^+^ CD11b^+^ CD11c^+^ and CD45^+^ CD11b^−^ CD11c^+^ populations in spleens of rTg4510 and WT mice at 9 months of age (Fig. 5). In the rTg4510 mouse cornea, CD45^+^ leukocyte frequency of live splenocytes was similar to age-matched WT mice (Fig. 5a, b). In the CD45^+^CD11b^+^ and CD45^+^CD11c^+^ populations, the percentages of CD86^+^ and CD80^+^ populations were similar between WT and rTg4510 mice (Fig. 5c). The comparative plots of absolute cell numbers demonstrate that there was a rise in the abundance of CD86^+^ and CD80^+^ cells in the CD11b^+^ splenocyte populations of the rTg4510 mice compared to WT mice (Fig. 5d). The splenic CD11c^+^ population, on the other hand, showed no notable differences in the size of CD86^+^ and CD80^+^ populations between WT and rTg4510 mice (Fig. 5d).

### Quantification of flow cytometry data

**Fig. 6:**
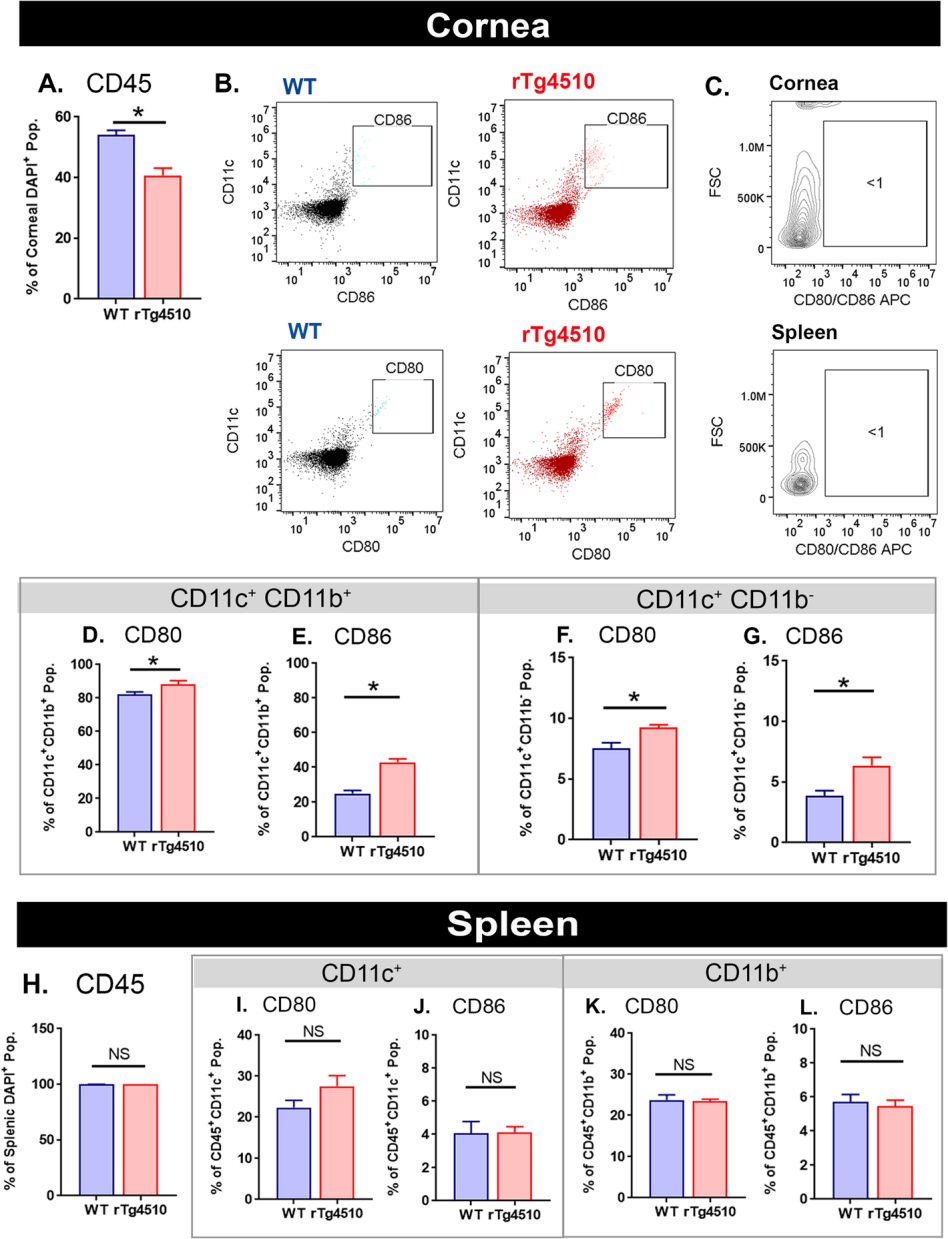
Quantification of cornea and spleen flow cytometry data in WT and rTg4510 cohorts. **a** There was a significantly lower frequency of CD45^+^ leukocytes in rTg4510 corneas compared to WT mice. **b** Representative dot plots demonstrate the shift in the “activated” DC phenotypes (CD80^+^ and CD86^+^) that are more prominent in rTg4510 cohorts. **c** Unstained samples of cornea and spleen tissues show the negative populations thus serving as a guide for identifying the positive population of CD80/CD86. **d**, **e** In the CD11c^+^CD11b^+^ corneal DC subset, the proportion of CD80^+^ and CD86^+^ DCs was reduced in WT mice compared to rTg4510 mice (*P* < 0.05). **f**, **g** Similar declines in the CD80^+^ and CD86^+^ DC populations were observed in CD11c^+^CD11b^−^ subset (*P* < 0.05). **h**–**l** Comparing WT and rTg4510 spleen tissues, no significant change in CD45, CD80, and CD86 populations was found in splenic CD11c^+^ or CD11b^+^ immune cell subsets (*P* > 0.05). Data are shown as mean ± SEM, where * indicates *P* ≤ 0.05 (*n* = 3 per group for corneas and *n* = 4 per group for spleens) as determined using the unpaired Student's *t*-test

In corneas, the proportion of CD45^+^ leukocytes was significantly lower in rTg4510 cohorts at 9 months of age compared to WT mice (Fig. 6a, *P* < 0.05). Representative dot plots demonstrate the shift towards an elevated population of CD11c^+^CD80^+^ and CD11c^+^ CD86^+^ in rTg4510 mice (Fig. 6b). The positive populations of CD80 and CD86 were then confirmed by the unstained control and isotype control antibodies, which depict the negative populations in both cornea and spleen tissues (Fig. 6c and Supplementary Fig. 2S). Among CD11c^+^CD11b^+^ DCs, rTg4510 mouse corneas had a significantly higher proportion of CD80^+^ and CD86^+^ “activated” populations compared to WT mice (Fig. 6d, e, *P* < 0.05). This shift towards the “activated” DC phenotypes in rTg4510 cohort was also significant in the “stromal” CD11c^+^CD11b^+^ DC subsets (Fig. 6f, g, *P* < 0.05). Among CD11c^+^ and CD11b^+^ immune cell subsets existing in the spleen, the proportion of CD45 leukocytes (Fig. 6h, *P* > 0.05), CD80^+^, and CD86^+^ DCs was not statistically different between WT and rTg4510 cohorts (Fig. 6i–l, *P* > 0.05).

### Gene expression of CaMKIIa in the mouse cornea

In this mouse model of tauopathy, tau transgene expression is dependent on the CaMKIIa promoter which drives transgene expression in the forebrain. We investigated whether CaMKIIa is expressed locally within the corneas of WT and rTg4510 (Supplementary Fig. 1S). There was no detectable expression of CaMKIIa in the corneas, as well as no significant difference in its expression between WT and rTg4510 cohorts at 4 months of age (Supplementary Fig. 1S, *P* > 0.05). This observation confirms that the corneal neuropathology was not driven by local tau transgene expression.

## Discussion

In this study, we characterized the temporal effects of CNS pathological tau accumulation on the corneal neuroimmune phenotype in mice. Our data demonstrate, for the first time, that corneal nerves and DCs are altered in a mouse model of tauopathy, indicating a peripheral manifestation of CNS tauopathy and/or neurodegeneration. Furthermore, in rTg4510 mice, corneal DCs displayed an altered immunophenotype (lower field area, total dendrite length, and number of dendrites per cell), a lower cell density, and an activated status. The differences in DC morphology were most apparent in the peripheral cornea, with the lower field area apparent by 6 months in rTg4510 mice, which occurred prior to the nerve differences. Lastly, there was a minimal effect of CNS tauopathy on the immunophenotype of analogous cell populations in the spleen. These unexpected findings highlight the possibility that examining corneal neuroimmune features could provide a novel strategy to detect the peripheral manifestations of CNS tauopathy, and further reflect the severity of the tau-driven neurodegenerative processes.

The axonal atrophy of neurons is a pathological hallmark of neurodegenerative conditions, such as AD [55, 56] and other tauopathies [57]. Tau that aggregates in neurons can propagate via cell-to-cell spreading, leading to the impairment of neuronal networks [58]. Our data, showing the temporal pattern of corneal nerve axonopathy in the presence of CNS tauopathy, suggest a PNS manifestation in rTg4510 mice that expresses forebrain-specific hyperphosphorylated tau. The occurrence of the corneal neuropathology coincided with the reported increasing levels of tau burden in the cortex and hippocampus at 8 months of age, along with severe brain atrophy and cognitive impairment at 12 months [6, 42]. Furthermore, our CaMKIIa gene expression data confirm that these corneal neuropathological phenotypes were not driven by local tau transgene expression, which is under the control of the CaMKIIa promoter, suggesting a likelihood of a secondary manifestation of CNS pathology. This concept of peripheral manifestation of CNS pathology is reported in the PS19Tg mouse model of tauopathy where mice develop motor dysfunction and sciatic nerve pathology, accompanied by a high abundance of mutated tau proteins in the sciatic nerves [59].

The mechanism underlying the impaired corneal nerves in rTg4510 mice is not entirely understood. In cross-sectional studies of individuals with PD, evidence of a decrement in the anisotropic component of white matter fibers in the early trigeminal nerve was detected by magnetic resonance imaging [60]. In addition, lower corneal nerve density and nerve length has been reported in individuals with PD [20, 35, 36]. Tau propagation is reported to depend on neural connectivity. For example, an abundance of hyperphosphorylated tau is reported in the brainstem and spinal cord [61]. While evidence for the propagation of misfolded tau proteins is emerging [61, 62], future studies are necessary to confirm the presence of abnormal tau in the trigeminal ganglion where the neuronal cell bodies of the corneal nerves are located and merge into a single, large sensory root entering the brainstem.

The alterations to corneal nerve architecture already identified clinically in PD [35, 36], MS [14, 15], and AD [16, 37], led us to postulate that, in addition to corneal neuropathology, corneal DCs might also be affected in the mouse model of tauopathy due to the known intimate neuroimmune crosstalk in the corneal epithelium. DCs secrete neurotrophins, such as ciliary neurotrophic factor, that regulate corneal sensory nerves during homeostasis and following nerve injury [47]. Pharmacological depletion of DCs in the mouse cornea has been shown to correlate with lower corneal nerve densities under homeostatic conditions [32] and following injury [47]. We found that the lower density of corneal nerve axons in the peripheral cornea paralleled a lower DC density in the mouse model of tauopathy. The gradual age-related increase in SBNP measurements in the aged WT mice may be explained by a true physiological increase in SBNP, as has been reported in rats [63]. However, other studies in mice report a decline in nerve density with aging [50, 64]. Furthermore, corneal nerve density differs according to strain [65]. In our study, all mice were on an Agouti background; thus, it is possible that this strain may have different nerve densities compared to the C57BL/6 strain. It is also worthwhile to note that a range of approaches have been used to measure corneal nerves, including thresholding [46, 47, 66], nerve tracing [46, 50], Scholl analysis [67], or a combination of manual tracing and thresholding [65]. This is an ongoing challenge in the literature, and thus while we acknowledge that thresholding has its limitations, we have controlled for this as best as possible by performing all immunostaining runs for age-matched cohorts in parallel.

The presence of corneal nerve beading in the rTg4510 aged cohort may indicate a nerve repair process, as has been described in individuals with dry eye disease [68], ultraviolet keratitis [69], and diabetes [70]. Nerve beading may indicate an abnormal accumulation of organelles that results in disrupted axonal transport [71]. Further studies are warranted to investigate the underlying mechanisms that drive corneal nerve axonopathy in the mouse model of tauopathy. Furthermore, measurements of physiological parameters, such as tear production, tear osmolarity, and corneal sensitivity, are of interest to determine if the described corneal nerve changes relate to altered ocular surface function.

In addition to direct tau-induced axonal impairment, the diminishing presence of corneal DCs may also impair the trophic support provided to the corneal nerves [57] in the rTg4510 mice. However, other studies have reported that higher corneal DC densities are associated with a lower nerve density in individuals with MS [14] and diabetes [72]. This discrepancy may be explained by the chronic systemic inflammation that occurs in these disease conditions. This systemic inflammation was not evident in our mouse model of tauopathy, as shown by the minimal effect of tauopathy on the population of activated splenic immune cell subsets.

Our study provides novel evidence of an altered corneal DC morphology and activated phenotype in the mouse model of pathological tau accumulation. Corneal DCs adopt different patterns in their distribution, density, and morphology under local and systemic inflammatory conditions in mice [51, 73]. Here, we report that the abnormalities in DC morphology reflect an activated phenotype in the transgenic mouse model of FTD. To interrogate the activation state of corneal DCs with a smaller field area, we focused on epithelial and stromal DC subsets [53] due to their central role in corneal immunity [74], their clinical relevance [48], and their capacity to undergo maturation through enhanced expression of activation markers, such as CD86 and CD80 in inflamed corneas [27, 73]. We found that the proportion of both CD86^+^ and CD80^+^ epithelial DCs were higher in tau transgenic mice, suggesting that morphological and density differences in the epithelial DCs were associated with upregulated cell surface expression of activation markers. In the context of CNS diseases, similar activation phenotypes have been reported in peripheral blood DCs from AD patients [75]. In double-transgenic amyloid precursor protein (APP)/presenilin 1 (PS1) murine models of AD, the role of DCs in amyloid plaque accumulation in the brain parenchyma is supported by evidence of increased formation of amyloid plaques following the systemic depletion of DCs [76]. Furthermore, the activated DCs engaged with T cells at sites of entry into the brain, presumably in response to the amyloid plaques [77, 78]. Although T cell infiltration of the cornea was not measured in this study, our data highlighting DC maturation (i.e., density, immunophenotype, and morphology) suggest a dysregulation in corneal immunity associated with the CNS degenerative disorders.

Recent evidence implicates that random insertions of the *MAPT* P301L and associated transgenes disrupt endogenous mouse genes and contribute to the neuropathological phenotypes in the rTg4510 mouse model [39]. Thus, it is relevant to examine the corneal nerves and DC phenotype in tau transgenic mice where transgene insertion has not disrupted endogenous genes (such as the PS19 tau transgenic mouse [39]), and also in APP/PS1 models to determine whether these neuroimmune changes also occur in related mouse models of dementia.

We also found subtle changes to the subpopulations of splenic immune cells (CD11b^+^ and CD11c^+^) in the presence of CNS tauopathy. Growing evidence supports a role for systemic inflammation as an exacerbating force in the pathogenesis of CNS degeneration (reviewed in [79, 80]), with misfolded proteins [81, 82], altered peripheral immune cells [83–85], and peripheral metabolic dysregulation [86] all reported. Here, the minor changes in the splenic CD86^+^ and CD80^+^CD11c^+^ populations and the elevation of CD86^+^ and CD80^+^CD11b^+^ populations in the rTg4510 mice suggest that the CD11b^+^ myeloid lineage cells may be altered in rTg4510 mice. This phenomenon is supported in part by the evidence from peripheral blood myeloid DCs in AD where the monocytic DCs have an increased expression of proinflammatory markers [75]. It is hypothesized that peripheral myeloid cells migrate to the CNS where they phagocytose tau aggregates or tau-laden neurons [87]; however, the contribution of peripheral myeloid cells to the pathogenesis of AD is still under debate.

## Conclusion

Our data show that not only were corneal nerves altered, but that epithelial DCs were phenotypically activated and exhibited morphological changes in the corneas from rTg4510 mice, suggesting a peripheral manifestation of the CNS tauopathy. The changes to epithelial DCs, followed by a loss of corneal nerve fibers, provide new insights into the effect of CNS tauopathy on the peripheral nervous system. These findings provide rationale for evaluating the diagnostic accuracy, including sensitivity and specificity, of corneal imaging of epithelial DC parameters as a disease marker in clinical populations with tauopathy.

## Supplementary information

**Additional file 1 Supplementary Fig 1S.** CaMKIIa gene expression in WT and rTg4510 mouse cornea at 3 months of age. Corneas were assessed for gene expression of CaMKIIa, which drives the tau transgene in rTg4510 mouse model. There was no detectable change in CaMKIIa gene expression between WT and rTg4510 cohorts (*P* > 0.05). Data are shown as mean ± SEM, where NS indicates no significant *P* > 0.05 (n = 11 for each genotype) as shown in the unpaired Student *t*-test.

**Additional file 2 Supplementary Fig 2S.** Gating strategies for CD80/CD86-APC and CD45-PE. **a** Histogram plot showing unstained, isotype control and positively stained population for CD80/CD86-APC antibody. **b** Negative populations from unstained and isotype control and positive population for CD45-PE antibody.

## Data Availability

The datasets used and/or analyzed during the current study are available from the corresponding authors on reasonable request

## Abbreviations

AD: Alzheimer’s disease
APP: Amyloid beta precursor proteins
CaMKII: Ca^2+^-calmodulin-dependent protein kinase II
CNS: Central nervous system
DCs: Dendritic cells
EDTA: Ethylenediaminetetraacetic acid
FTD: Frontotemporal dementia
MS: Multiple sclerosis
MCI: Mild cognitive impairment
OCT: Optical coherence tomography
PD: Parkinson’s disease
PNS: Peripheral nervous system
PS1: Presenilin 1
rTg4510: Transgenic tauopathy mouse model
SBNP: Sub-basal nerve plexus
SD-OCT: Spectral domain optical coherence tomography
SNT: Superficial nerve terminals
WT: Wild type

## References

[1] Stamer K, Vogel R, Thies E, Mandelkow E, Mandelkow EM (2002). Tau blocks traffic of organelles, neurofilaments, and APP vesicles in neurons and enhances oxidative stress. J Cell Biol..

[2] Hoover BR, Reed MN, Su J, Penrod RD, Kotilinek LA, Grant MK (2010). Tau mislocalization to dendritic spines mediates synaptic dysfunction independently of neurodegeneration. Neuron..

[3] Kopeikina KJ, Polydoro M, Tai HC, Yaeger E, Carlson GA, Pitstick R (2013). Synaptic alterations in the rTg4510 mouse model of tauopathy. J Comp Neurol..

[4] Santacruz K, Lewis J, Spires T, Paulson J, Kotilinek L, Ingelsson M (2005). Tau suppression in a neurodegenerative mouse model improves memory function. Science..

[5] Yoshiyama Y, Higuchi M, Zhang B, Huang SM, Iwata N, Saido TC (2007). Synapse loss and microglial activation precede tangles in a P301S tauopathy mouse model. Neuron..

[6] Spires TL, Orne JD, SantaCruz K, Pitstick R, Carlson GA, Ashe KH (2006). Region-specific dissociation of neuronal loss and neurofibrillary pathology in a mouse model of tauopathy. Am J Pathol..

[7] Colligris P, Perez de Lara MJ, Colligris B, Pintor J (2018). Ocular manifestations of Alzheimer’s and other neurodegenerative diseases: the prospect of the eye as a tool for the early diagnosis of Alzheimer’s disease. J Ophthalmol.

[8] Moreno-Ramos T, Benito-Leon J, Villarejo A, Bermejo-Pareja F (2013). Retinal nerve fiber layer thinning in dementia associated with Parkinson’s disease, dementia with Lewy bodies, and Alzheimer’s disease. J Alzheimers Dis..

[9] Cheung CY, Ong YT, Hilal S, Ikram MK, Low S, Ong YL (2015). Retinal ganglion cell analysis using high-definition optical coherence tomography in patients with mild cognitive impairment and Alzheimer’s disease. J Alzheimers Dis..

[10] Marziani E, Pomati S, Ramolfo P, Cigada M, Giani A, Mariani C (2013). Evaluation of retinal nerve fiber layer and ganglion cell layer thickness in Alzheimer’s disease using spectral-domain optical coherence tomography. Invest Ophth Vis Sci..

[11] Chiasseu M, Alarcon-Martinez L, Belforte N, Quintero H, Dotigny F, Destroismaisons L (2017). Tau accumulation in the retina promotes early neuronal dysfunction and precedes brain pathology in a mouse model of Alzheimer’s disease. Molecular Neurodegeneration..

[12] Grimaldi A, Brighi C, Peruzzi G, Ragozzino D, Bonanni V, Limatola C (2018). Inflammation, neurodegeneration and protein aggregation in the retina as ocular biomarkers for Alzheimer’s disease in the 3xTg-AD mouse model. Cell Death Dis..

[13] Harrison IF, Whitaker R, Bertelli PM, O’Callaghan JM, Csincsik L, Bocchetta M (2019). Optic nerve thinning and neurosensory retinal degeneration in the rTg4510 mouse model of frontotemporal dementia. Acta Neuropathologica Communications..

[14] Bitirgen G, Akpinar Z, Malik RA, Ozkagnici A (2017). Use of corneal confocal microscopy to detect corneal nerve loss and increased dendritic cells in patients with multiple sclerosis. JAMA Ophthalmol..

[15] Petropoulos IN, Kamran S, Li Y, Khan A, Ponirakis G, Akhtar N (2017). Corneal confocal microscopy: an imaging endpoint for axonal degeneration in multiple sclerosis. Invest Ophthalmol Vis Sci..

[16] Ponirakis G, Al Hamad H, Sankaranarayanan A, Khan A, Chandran M, Ramadan M (2019). Association of corneal nerve fiber measures with cognitive function in dementia. Ann Clin Transl Neurol..

[17] Bucher F, Schneider C, Blau T, Cursiefen C, Fink GR, Lehmann HC (2015). Small-fiber neuropathy is associated with corneal nerve and dendritic cell alterations: an in vivo confocal microscopy study. Cornea..

[18] Vieira L, Anjos R, De Sousa A, Silva N, Basilio AL, Maduro VS (2015). Peripheral neuropathy in Parkinson’s disease: an in vivo confocal microscopy study. Eur J Neurol.

[19] Mikolajczak J, Zimmermann H, Kheirkhah A, Kadas EM, Oberwahrenbrock T, Muller R (2017). Patients with multiple sclerosis demonstrate reduced subbasal corneal nerve fibre density. Mult Scler J..

[20] Podgorny PJ, Suchowersky O, Romanchuk KG, Feasby TE (2016). Evidence for small fiber neuropathy in early Parkinson’s disease. Parkinsonism Relat D..

[21] Marfurt CF, Cox J, Deek S, Dvorscak L (2010). Anatomy of the human corneal innervation. Exp Eye Res..

[22] He J, Bazan HE (2016). Neuroanatomy and neurochemistry of mouse cornea. Invest Ophthalmol Vis Sci..

[23] Rozsa AJ, Beuerman RW (1982). Density and organization of free nerve endings in the corneal epithelium of the rabbit. Pain..

[24] Al-Aqaba MA, Dhillon VK, Mohammed I, Said DG, Dua HS (2019). Corneal nerves in health and disease. Prog Retin Eye Res.

[25] Yamagami S, Yokoo S, Usui T, Yamagami H, Amano S, Ebihara N (2005). Distinct populations of dendritic cells in the normal human donor corneal epithelium. Invest Ophthalmol Vis Sci..

[26] Mastropasqua L, Nubile M, Lanzini M, Carpineto P, Ciancaglini M, Pannellini T (2006). Epithelial dendritic cell distribution in normal and inflamed human cornea: in vivo confocal microscopy study. Am J Ophthalmol..

[27] Hamrah P, Zhang Q, Liu Y, Dana MR (2002). Novel characterization of MHC class II-negative population of resident corneal Langerhans cell-type dendritic cells. Invest Ophthalmol Vis Sci..

[28] Knickelbein JE, Watkins SC, McMenamin PG, Hendricks RL (2009). Stratification of antigen-presenting cells within the normal cornea. Ophthalmol Eye Dis..

[29] Hamrah P, Huq SO, Liu Y, Zhang Q, Dana MR (2003). Corneal immunity is mediated by heterogeneous population of antigen-presenting cells. J Leukocyte Biol..

[30] Hattori T, Takahashi H, Dana R (2016). Novel insights into the immunoregulatory function and localization of dendritic cells. Cornea..

[31] Steinman RM, Hemmi H (2006). Dendritic cells: translating innate to adaptive immunity. Curr Top Microbiol Immunol..

[32] Gao N, Lee P, Yu FS (2016). Intraepithelial dendritic cells and sensory nerves are structurally associated and functional interdependent in the cornea. Sci Rep..

[33] Cruzat A, Qazi Y, Hamrah P (2017). In vivo confocal microscopy of corneal nerves in health and disease. Ocul Surf..

[34] De Silva MEH, Zhang AC, Karahalios A, Chinnery HR, Downie LE (2017). Laser scanning in vivo confocal microscopy (IVCM) for evaluating human corneal sub-basal nerve plexus parameters: protocol for a systematic review. BMJ Open..

[35] Kass-Iliyya L, Javed S, Gosal D, Kobylecki C, Marshall A, Petropoulos IN (2015). Small fiber neuropathy in Parkinson’s disease: a clinical, pathological and corneal confocal microscopy study. Parkinsonism Relat Disord..

[36] Misra SL, Kersten HM, Roxburgh RH, Danesh-Meyer HV, McGhee CN (2017). Corneal nerve microstructure in Parkinson’s disease. J Clin Neurosci..

[37] Ornek N, Dag E, Ornek K (2015). Corneal sensitivity and tear function in neurodegenerative diseases. Curr Eye Res..

[38] Goodwin LO, Splinter E, Davis TL, Urban R, He H, Braun RE (2019). Large-scale discovery of mouse transgenic integration sites reveals frequent structural variation and insertional mutagenesis. Genome Res..

[39] Gamache J, Benzow K, Forster C, Kemper L, Hlynialuk C, Furrow E (2019). Factors other than hTau overexpression that contribute to tauopathy-like phenotype in rTg4510 mice. Nat Commun..

[40] Menkes-Caspi N, Yamin HG, Kellner V, Spires-Jones TL, Cohen D, Stern EA (2015). Pathological tau disrupts ongoing network activity. Neuron..

[41] Ramsden M, Kotilinek L, Forster C, Paulson J, McGowan E, SantaCruz K (2005). Age-dependent neurofibrillary tangle formation, neuron loss, and memory impairment in a mouse model of human tauopathy (P301L). J Neurosci..

[42] Blackmore T, Meftah S, Murray TK, Craig PJ, Blockeel A, Phillips K (2017). Tracking progressive pathological and functional decline in the rTg4510 mouse model of tauopathy. Alzheimer's Research & Therapy..

[43] Bailey RM, Howard J, Knight J, Sahara N, Dickson DW, Lewis J (2014). Effects of the C57BL/6 strain background on tauopathy progression in the rTg4510 mouse model. Mol Neurodegener..

[44] Hodges JR, Davies RR, Xuereb JH, Casey B, Broe M, Bak TH (2004). Clinicopathological correlates in frontotemporal dementia. Ann Neurol..

[45] Downie LE, Stainer MJ, Chinnery HR (2014). Monitoring of strain-dependent responsiveness to TLR activation in the mouse anterior segment using SD-OCT. Invest Ophthalmol Vis Sci..

[46] Downie LE, Naranjo Golborne C, Chen M, Ho N, Hoac C, Liyanapathirana D (2018). Recovery of the sub-basal nerve plexus and superficial nerve terminals after corneal epithelial injury in mice. Exp Eye Res..

[47] Gao N, Yan C, Lee P, Sun H, Yu FS (2016). Dendritic cell dysfunction and diabetic sensory neuropathy in the cornea. J Clin Invest..

[48] Kheirkhah A, Rahimi Darabad R, Cruzat A, Hajrasouliha AR, Witkin D, Wong N (2015). Corneal epithelial immune dendritic cell alterations in subtypes of dry eye disease: a pilot in vivo confocal microscopic study. Invest Ophthalmol Vis Sci..

[49] Lagali NS, Badian RA, Liu X, Feldreich TR, Arnlov J, Utheim TP (2018). Dendritic cell maturation in the corneal epithelium with onset of type 2 diabetes is associated with tumor necrosis factor receptor superfamily member 9. Sci Rep..

[50] De Silva MEH, Hill LJ, Downie LE, Chinnery HR (2019). The effects of aging on corneal and ocular surface homeostasis in mice. Invest Ophthalmol Vis Sci..

[51] Jiao H, Naranjo Golborne C, Dando SJ, McMenamin PG, Downie LE, Chinnery HR. Topographical and morphological differences of corneal dendritic cells during steady state and inflammation. Ocul Immunol Inflamm. 2019:1–10. 10.1080/09273948.2019.1646775 31429614

[52] Gu BJ, Sun C, Fuller S, Skarratt KK, Petrou S, Wiley JS (2014). A quantitative method for measuring innate phagocytosis by human monocytes using real-time flow cytometry. Cytometry A..

[53] Hattori T, Chauhan SK, Lee H, Ueno H, Dana R, Kaplan DH (2011). Characterization of Langerin-expressing dendritic cell subsets in the normal cornea. Invest Ophthalmol Vis Sci..

[54] Lee HS, Amouzegar A, Dana R (2017). Kinetics of corneal antigen presenting cells in experimental dry eye disease. BMJ Open Ophthalmol..

[55] Geula C, Nagykery N, Nicholas A, Wu CK (2008). Cholinergic neuronal and axonal abnormalities are present early in aging and in Alzheimer disease. J Neuropathol Exp Neurol..

[56] Zhu B, Luo L, Moore GR, Paty DW, Cynader MS (2003). Dendritic and synaptic pathology in experimental autoimmune encephalomyelitis. Am J Pathol..

[57] Kanaan NM, Morfini GA, LaPointe NE, Pigino GF, Patterson KR, Song Y (2011). Pathogenic forms of tau inhibit kinesin-dependent axonal transport through a mechanism involving activation of axonal phosphotransferases. J Neurosci..

[58] Goedert M, Eisenberg DS, Crowther RA (2017). Propagation of tau aggregates and neurodegeneration. Annu Rev Neurosci..

[59] Merchan-Rubira J, Sebastian-Serrano A, Diaz-Hernandez M, Avila J, Hernandez F (2019). Peripheral nervous system effects in the PS19 tau transgenic mouse model of tauopathy. Neurosci Lett..

[60] Arrigo A, Rania L, Calamuneri A, Postorino EI, Mormina E, Gaeta M (2018). Early corneal innervation and trigeminal alterations in Parkinson disease: a pilot study. Cornea..

[61] Ahmed Z, Cooper J, Murray TK, Garn K, McNaughton E, Clarke H (2014). A novel in vivo model of tau propagation with rapid and progressive neurofibrillary tangle pathology: the pattern of spread is determined by connectivity, not proximity. Acta Neuropathol..

[62] Hallinan GI, Vargas-Caballero M, West J, Deinhardt K (2019). Tau misfolding efficiently propagates between individual intact hippocampal neurons. J Neurosci..

[63] Dvorscak L, Marfurt CF (2008). Age-related changes in rat corneal epithelial nerve density. Invest Ophthalmol Vis Sci..

[64] Stepp MA, Pal-Ghosh S, Tadvalkar G, Williams A, Pflugfelder SC, de Paiva CS (2018). Reduced intraepithelial corneal nerve density and sensitivity accompany desiccating stress and aging in C57BL/6 mice. Exp Eye Res..

[65] Pham TL, Kakazu A, He J, Bazan HEP (2019). Mouse strains and sexual divergence in corneal innervation and nerve regeneration. FASEB J..

[66] Chucair-Elliott AJ, Zheng M, Carr DJ (2015). Degeneration and regeneration of corneal nerves in response to HSV-1 infection. Invest Ophthalmol Vis Sci..

[67] Stepp MA, Pal-Ghosh S, Tadvalkar G, Li L, Brooks SR, Morasso MI (2018). Molecular basis of Mitomycin C enhanced corneal sensory nerve repair after debridement wounding. Sci Rep..

[68] Benitez-Del-Castillo JM, Acosta MC, Wassfi MA, Diaz-Valle D, Gegundez JA, Fernandez C (2007). Relation between corneal innervation with confocal microscopy and corneal sensitivity with noncontact esthesiometry in patients with dry eye. Invest Ophthalmol Vis Sci..

[69] Abedi F, Hamrah P (2018). Corneal subbasal nerve recovery in an acute case of ultraviolet keratitis treated with autologous serum eye drops. J Ophthalmol..

[70] Ishibashi F, Kojima R, Taniguchi M, Kosaka A, Uetake H, Tavakoli M (2016). The expanded bead size of corneal C-nerve fibers visualized by corneal confocal microscopy is associated with slow conduction velocity of the peripheral nerves in patients with type 2 diabetes mellitus. J Diabetes Res..

[71] Stokin GB, Lillo C, Falzone TL, Brusch RG, Rockenstein E, Mount SL (2005). Axonopathy and transport deficits early in the pathogenesis of Alzheimer’s disease. Science..

[72] Leppin K, Behrendt AK, Reichard M, Stachs O, Guthoff RF, Baltrusch S (2014). Diabetes mellitus leads to accumulation of dendritic cells and nerve fiber damage of the subbasal nerve plexus in the cornea. Invest Ophthalmol Vis Sci..

[73] Hamrah P, Liu Y, Zhang Q, Dana MR (2003). Alterations in corneal stromal dendritic cell phenotype and distribution in inflammation. Arch Ophthalmol..

[74] Forrester JV, Xu H, Kuffova L, Dick AD, McMenamin PG (2010). Dendritic cell physiology and function in the eye. Immunol Rev..

[75] Ciaramella A, Bizzoni F, Salani F, Vanni D, Spalletta G, Sanarico N (2010). Increased pro-inflammatory response by dendritic cells from patients with Alzheimer’s disease. J Alzheimers Dis..

[76] Butovsky O, Kunis G, Koronyo-Hamaoui M, Schwartz M (2007). Selective ablation of bone marrow-derived dendritic cells increases amyloid plaques in a mouse Alzheimer’s disease model. Eur J Neurosci..

[77] Fisher Y, Nemirovsky A, Baron R, Monsonego A (2011). Dendritic cells regulate amyloid-beta-specific T-cell entry into the brain: the role of perivascular amyloid-beta. J Alzheimers Dis..

[78] Monsonego A, Imitola J, Petrovic S, Zota V, Nemirovsky A, Baron R (2006). Aβ-induced meningoencephalitis is IFN-γ-dependent and is associated with T cell-dependent clearance of Aβ in a mouse model of Alzheimer’s disease. Proceedings of the National Academy of Sciences of the United States of America..

[79] Cao W, Zheng H (2018). Peripheral immune system in aging and Alzheimer’s disease. Mol Neurodegener..

[80] Dionisio-Santos DA, Olschowka JA, O'Banion MK (2019). Exploiting microglial and peripheral immune cell crosstalk to treat Alzheimer’s disease. J Neuroinflammation..

[81] Dugger BN, Hoffman BR, Scroggins A, Serrano GE, Adler CH, Shill HA (2018). Tau immunoreactivity in peripheral tissues of human aging and select tauopathies. Neurosci Lett..

[82] Mattsson N, Zetterberg H, Janelidze S, Insel PS, Andreasson U, Stomrud E (2016). Plasma tau in Alzheimer disease. Neurology..

[83] Simard AR, Soulet D, Gowing G, Julien JP, Rivest S (2006). Bone marrow-derived microglia play a critical role in restricting senile plaque formation in Alzheimer’s disease. Neuron..

[84] Merlini M, Kirabali T, Kulic L, Nitsch RM, Ferretti MT (2018). Extravascular CD3+ T cells in brains of Alzheimer disease patients correlate with tau but not with amyloid pathology: an immunohistochemical study. Neurodegener Dis..

[85] St-Amour I, Bosoi CR, Paré I, Ignatius Arokia Doss PM, Rangachari M, Hébert SS (2019). Peripheral adaptive immunity of the triple transgenic mouse model of Alzheimer’s disease. Journal of Neuroinflammation.

[86] Clarke JR, Lyra ESNM, Figueiredo CP, Frozza RL, Ledo JH, Beckman D (2015). Alzheimer-associated Abeta oligomers impact the central nervous system to induce peripheral metabolic deregulation. EMBO Mol Med..

[87] Prokop S, Miller KR, Drost N, Handrick S, Mathur V, Luo J (2015). Impact of peripheral myeloid cells on amyloid-beta pathology in Alzheimer’s disease-like mice. J Exp Med..

