# Supplementary figures and images for: Novel alterations in corneal neuroimmune phenotypes in mice with central nervous system tauopathy
Source: J Neuroinflammation. 2020 Apr 28;17:136. doi: 10.1186/s12974-020-01803-7 (PMC7189727; doi:10.1186/s12974-020-01803-7)

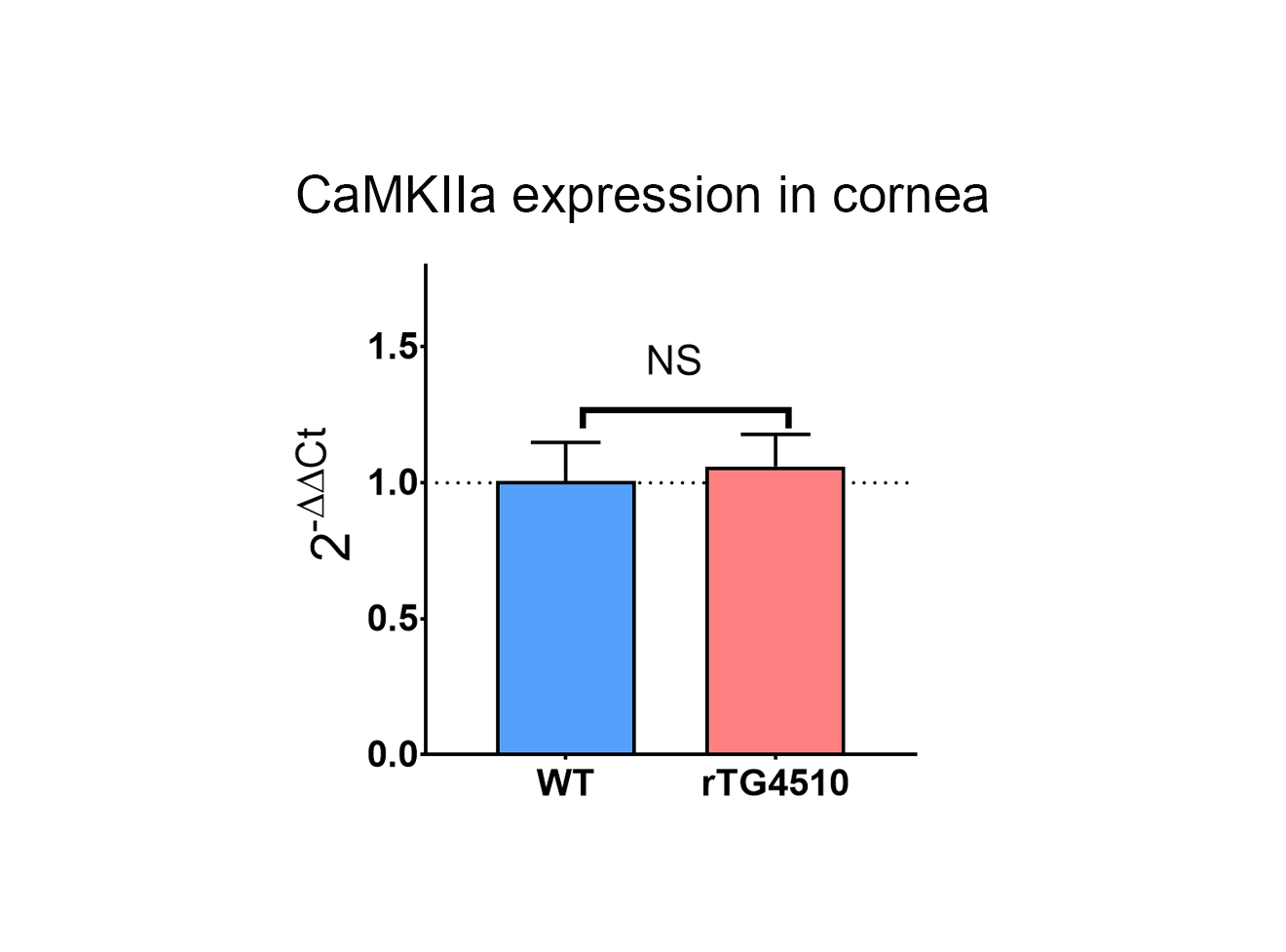

Supplement: Supplementary file 1 — Additional file 1 Supplementary Fig 1S. CaMKIIa gene expression in WT and rTg4510 mouse cornea at 3 months of age. Corneas were assessed for gene expression of CaMKIIa, which drives the tau transgene in rTg4510 mouse model. There was no detectable change in CaMKIIa gene expression between WT and rTg4510 cohorts (P > 0.05). Data are shown as mean ± SEM, where NS indicates no significant P > 0.05 (n = 11 for each genotype) as shown in the unpaired Student t-test. [file 12974_2020_1803_MOESM1_ESM.tif]

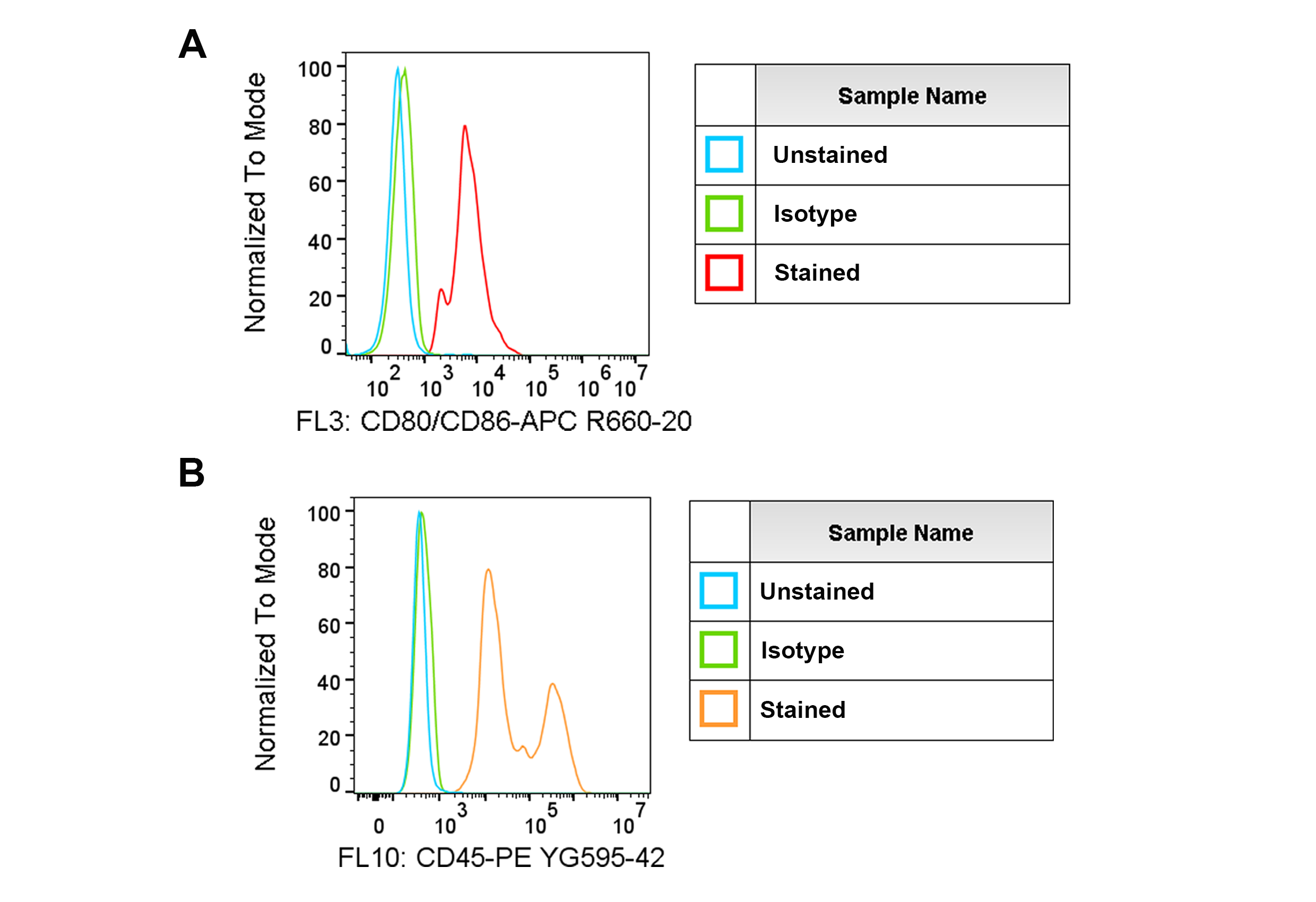

Supplement: Supplementary file 2 — Additional file 2 Supplementary Fig 2S. Gating strategies for CD80/CD86-APC and CD45-PE. a Histogram plot showing unstained, isotype control and positively stained population for CD80/CD86-APC antibody. b Negative populations from unstained and isotype control and positive population for CD45-PE antibody. [file 12974_2020_1803_MOESM2_ESM.tif]
